# Quantitative Analysis of Genomic DNA Degradation of *E. coli* Using Automated Gel Electrophoresis under Various Levels of Microwave Exposure

**DOI:** 10.3390/gels10040242

**Published:** 2024-04-02

**Authors:** Aditya Pandey, Omeed Momeni, Pramod Pandey

**Affiliations:** 1 Department of Electrical and Computer Engineering, University of California at Davis, Davis, CA 95616, USA; adiey@ucdavis.edu (A.P.); omomeni@ucdavis.edu (O.M.); 2 Department of Population Health and Reproduction, University of California at Davis, Davis, CA 95616, USA

**Keywords:** automated gel electrophoresis, *E. coli*, genomic DNA, microwave, degradation

## Abstract

The problem that this study addresses is to understand how microwave radiation is able to degrade genomic DNA of *E. coli*. In addition, a comparative study was made to evaluate the suitability of a high-throughput automated electrophoresis platform for quantifying the DNA degradation under microwave radiation. Overall, this study investigated the genomic DNA degradation of *E. coli* under microwave radiation using automated gel electrophoresis. To examine the viable organisms and degradation of genomic DNA under microwave exposure, we used three methods: (1) post-microwave exposure, where *E. coli* was enumerated using modified mTEC agar method using membrane filtration technique; (2) extracted genomic DNA of microwaved sample was quantified using the Qubit method; and (3) automated gel electrophoresis, the TapeStation 4200, was used to examine the bands of extracted DNA of microwaved samples. In addition, to examine the impacts of microwaves, *E. coli* colonies were isolated from a fecal sample (dairy cow manure), these colonies were grown overnight to prepare fresh *E. coli* culture, and this culture was exposed to microwave radiation for three durations: (1) 2 min; (2) 5 min; and (3) 8 min. In general, Qubit values (ng/µL) were proportional to the results of automated gel electrophoresis, TapeStation 4200, DNA integrity numbers (DINs). Samples from exposure studies (2 min, 5 min, and 8 min) showed no viable *E. coli*. Initial *E. coli* levels (at 0 min microwave exposure) were 5 × 10^8^ CFU/mL, and the *E. coli* level was reduced to a non-detectable level within 2 min of microwave exposure. The relationships between Qubit and TapeStation measurements was linear, except for when the DNA level was lower than 2 ng/µL. In 8 min of microwave exposure, *E. coli* DNA integrity was reduced by 61.7%, and DNA concentration was reduced by 81.6%. The overall conclusion of this study is that microwave radiation had a significant impact on the genomic DNA of *E. coli*, and prolonged exposure of *E. coli* to microwaves can thus lead to a loss of genomic DNA integrity and DNA concentrations.

## 1. Introduction

Separation, identification, and analysis of DNA fragments play a crucial role for determining environmental microbial pollutants. Gel electrophoresis based on agarose gel is considered to be the most effective method of separating DNA fragments, and it can analyze DNA fragments of varying sizes [1]. In general, electrophoresis of DNA is used for separating DNA fragments based on DNA sizes [2,3,4]; however, conventional gel electrophoresis is limited in throughput and requires a substantial amount of time and skills to monitor DNA fragments based on their size [5,6]. In conventional gel electrophoresis based on agarose gel, the DNA sample is loaded into pre-cast wells, and a current is applied to assist DNA fragment migration in agarose gel. Due to the negative charge in the phosphate backbone of the DNA, the fragments of DNA move towards positive charge under an electric field [1,6]. The migration rate of DNA fragments in agarose gel is based on the size of DNA molecules, agarose concentration, voltage applied, presence of ethidium bromide, and buffer [1,7,8,9]. 

In this study, we used a high-throughput method to understand DNA degradation under microwave exposure. Recent advances in gel electrophoresis and analytical software of image analysis can provide an alternative to conventional gel electrophoresis. For example, the TapeStation is a rapid and high-throughput method for analyzing DNA fragments based on their size, and it is highly sensitive, separates different sized fragments by automated electrophoreses, and provides an option for high sensitivity and high throughput [10]. Currently, many methods such as Qubit fluorometers, conventional gel electrophoresis, qPCR, pulsed field gel electrophoresis, and bioanalyzers are used for assessing genomic DNA fragments. To quantify DNA, Qubit fluorometers determine DNA based on the fluorescence intensity of fluorescent dye that binds to double-stranded DNA [11]. Qubit fluorometers allow for the sensitive quantification of DNA [12,13]. Bioanalyzers are used to quantify DNA; however, the application of bioanalyzers requires advanced skills [14,15]. Bioanalyzer platforms are used for a rapid, accurate, and cost-efficient quantification of DNA [16,17,18]. In the TapeStation, a high-throughput method, the process of gel electrophoresis is automated, and the automation of this process provides high-quality gel images and the analysis of bands in a relatively short time [19,20]. 

The main objectives of this study were to determine the level and type of DNA degradation under microwave radiation. To examine the impacts of microwave exposure on DNA integrity and DNA concentrations of *E. coli*, a series of experiments using environmental samples were conducted, and multiple methods and exposure times were tested. Overall, the goal of this study was to develop a simplified workflow and utilize automated gel electrophoresis to determine the impacts of microwave radiation on genomic DNA integrity and DNA reduction. We isolated *E. coli* from dairy manure, and *E. coli* was exposed to microwave radiation for various exposure times. Subsequently, genomic DNA was extracted from microwave-exposed *E. coli* samples. Recently, there has been a substantial renewed interest in the use of microwaves for the elimination of bacteria from food products, for food safety, and for microwave-based sterilization and heating of food products. For example, attempts are made to apply a microwave-assisted sterilization process for producing pathogen-free food products; however, additional studies are needed to understand the impacts of microwaves on the viability of bacteria and bacteria DNA degradation. Further, attempts are made to apply microwave radiation for treating wastewater and waste sludge [21,22]. 

## 2. Results and Discussion

### 2.1. Automated Electrophoresis and Electropherograms Using Automated Electrophoresis under Various Microwave Exposure Conditions

**Figure 1:**
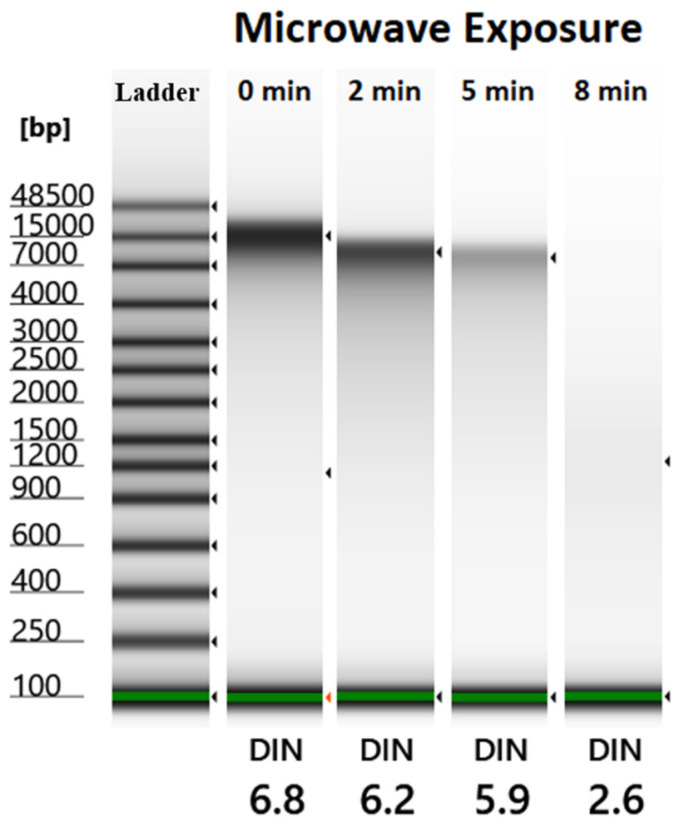
Results of automated gel electrophoresis image analysis using TapeStation 4200 under various levels of microwave radiation exposure time. Bands of the ladder are shown in the extreme left, and bands shown afterward (right side) are for different exposure times (0 min, 2 min, 5 min, and 8 min).

To evaluate the impacts of microwaves on bacteria degradation and DNA quality, a set of microwave exposure conditions were created. The experiments were performed in lab conditions using environmental samples, and test results were evaluated using a series of advanced instrumentations. The results of automated electrophoresis are shown in Figure 1, which describes the genomic DNA bands of microwave-exposed samples, corresponding DNA separation, and DNA integrity. The gel image of the genomic DNA of *E. coli* clearly shows a shift in DNA size, DNA distribution with increasing microwave exposure time, and subsequent degradation of genomic DNA. In lane 1, ladder bands of microwaved samples are shown, and lane 2 shows undigested (without microwave exposure) *E. coli* DNA. Lane 2, lane 3, and lane 4 show the DNA of *E. coli* samples exposed to microwave radiation for 0 min, 2 min, and 5 min, respectively. In the 8 min exposure time (result shown in lane 5), *E. coli* was degraded completely, and extracted DNA showed no bands. In the undigested (without microwaves) *E. coli* sample, genomic DNA is shown in band 1 (≈15,000 bp). In the *E. coli* sample which was exposed for 2 min, the genomic DNA band was lighter and fainter. In the 5 min exposure time, the genomic DNA band was further fainter. After 8 min of microwave exposure time, the extracted genomic DNA of samples showed no bands. In contrast, in the first three samples (0 min, 2 min, and 5 min exposure time), genomic DNA showed up as a single band. Without microwave exposure, the genomic DNA of *E. coli* showed a distinct band; however, no distinct genomic DNA band was visible on samples which were exposed to microwave radiation for 8 min. 

To evaluate the microwave exposure study results, we determined the integrity of genomic DNA using the DNA integrity number (i.e., DIN), a numerical value of the integrity of genomic DNA. During the microwave exposure study, a highly intact genomic DNA band was obtained for the sample without microwave exposure (control sample). Figure 1 shows the gel image with the DIN on the bottom of each gel lane. In general, the DIN value ranges between 1 and 10, where 10 represents highly intact genomic DNA and 1 indicates highly degraded genomic DNA. The DIN value was 6.8 for the genomic DNA of *E. coli* without microwave exposure. In the 2 min exposure, the integrity of genomic DNA was reduced by 8.8%, and in the 5 min microwave exposure, the integrity of genomic DNA was reduced by 13%. During 8 min of microwave exposure, the integrity of genomic DNA was reduced by 61.7%. 

**Figure 2:**
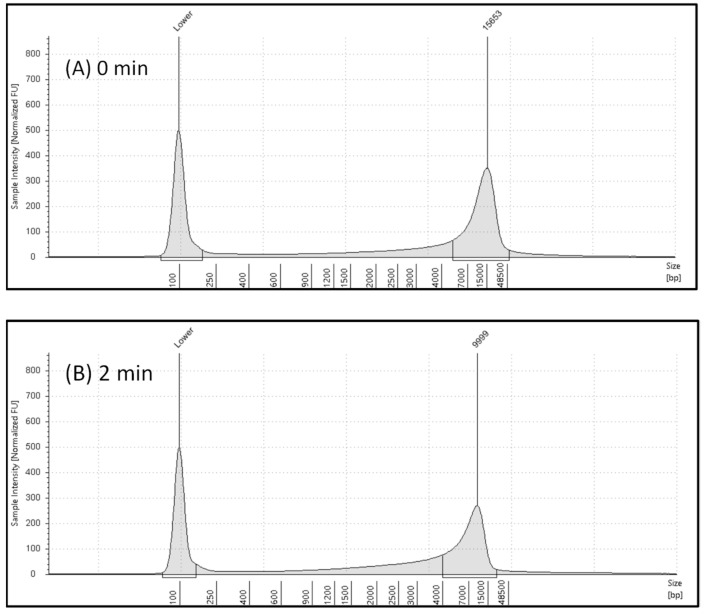
The analysis of electropherograms of the microwave exposure study using TapeStation 4200: (**A**) electropherogram of DNA sample without microwave exposure; (**B**) electropherogram of DNA under 2 min of microwave exposure. The reduction in DNA bp size is compared (0 min and 2 min). The genomic DNA bp size at 2 min was reduced from 15,653 to 9999 (36% lower than bp of genomic DNA of *E. coli* without microwave exposure).

**Figure 3:**
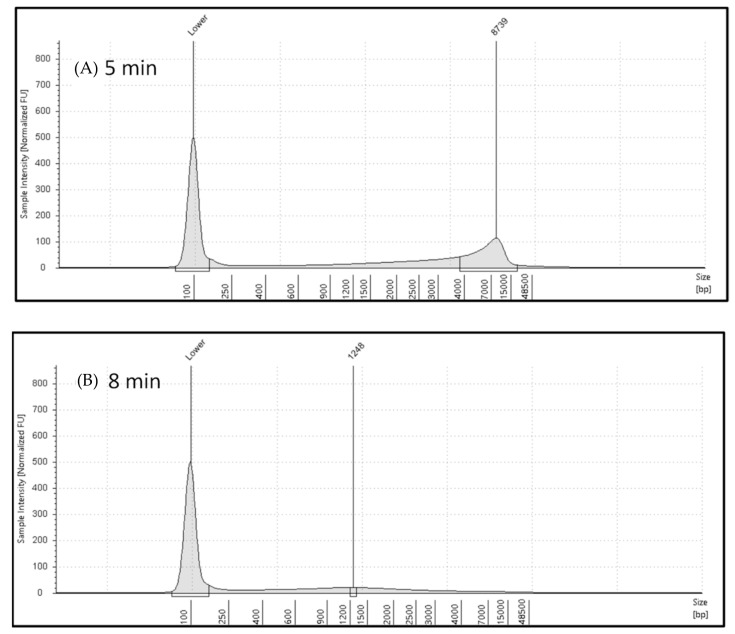
The results of the microwave exposure study by analyzing electropherograms using TapeStation 4200: (**A**) electropherogram of DNA sample for 5 min microwave exposure; (**B**) electropherogram of DNA for 8 min microwave exposure. Degradation of genomic DNA is compared between 5 min and 8 min. The genomic DNA bp size was reduced from 15,653 to 8739 in 5 min exposure (44% reduction), and it was further reduced from 15,653 to 1248 in 8 min (reduction by 92% compared to sample without microwave exposure).

The electropherogram of genomic DNA of *E. coli* without microwave exposure is shown in Figure 2A, which reflects a well-defined peak slightly below the largest ladder peak at 48,500 bp. With increasing microwave exposure, genomic DNA was degraded, genomic DNA migrated slightly below 15,000 bp, and this shift was observed in the electropherogram shown in Figure 2 (2 min exposure time). This observation is also reflected in Figure 3, which showed electropherograms for extended microwave exposure (5 min and 8 min). The sample with the highest DNA integrity has the highest DIN value of 6.8, and increasing microwave exposure resulted in decreasing DIN. Similar observations were observed in electropherograms shown in Figure 2 and Figure 3. The right-side peak in the electropherogram (Figure 2) showed 15,653 pb in the 0 min exposure and it was reduced to 9999 in the 2 min exposure. The bp size was decreased by 36% in the 2 min exposure compared to the genomic DNA of *E. coli* without microwave exposure. The genomic DNA bp size was reduced from 15,653 to 8739 in the 5 min exposure. Further microwave exposure resulted in an additional reduction in bp. For example, genomic DNA of *E. coli* showed a 1248 pb size in samples which were exposed for 8 min. About 92% DNA bp reduction occurred in 8 min microwave exposure (Figure 3). 

### 2.2. E. coli Enumeration and Corresponding Automated Electropherogram Analysis

**Figure 4:**
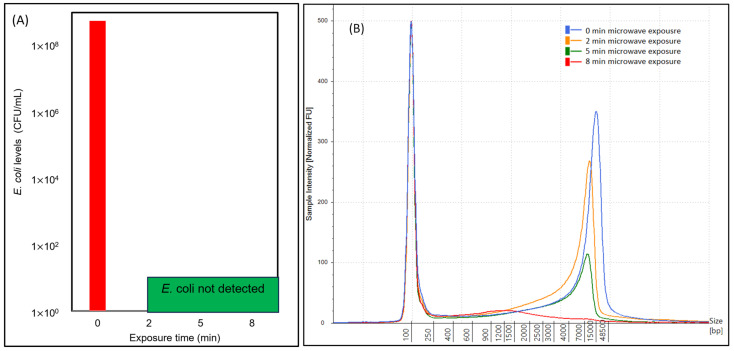
Results of *E. coli* enumeration and corresponding DNA intensity of microwave-exposed samples. Left figure (**A**) shows results of *E. coli* enumeration using modified mTEC agar, and right-side figure (**B**) shows corresponding DNA concentrations using TapeStation 4200. The comparative results are shown for multiple exposure times (0 min, 2 min, 5 min, and 8 min) in (**A**,**B**).

During each exposure time (0 min, 2 min, 5 min, and 8 min), samples were plated using the membrane filtration technique on modified mTEC agar, and *E. coli* colonies were enumerated. The *E. coli* levels (CFU/mL) for samples are shown in Figure 4A. Results showed that the *E. coli* level in the sample without microwave exposure was 5.6 × 10^8^ CFU/mL. When *E. coli* samples were exposed to microwaves for 2 min, the sample showed no recovery and growth of *E. coli*. Further, *E. coli* samples after 5 min and 8 min exposure were plated, and overnight incubation showed no *E. coli* recovery in modified mTEC agar. This reflects that 2 min microwave exposure damaged the *E. coli*, which resulted in no recovery in growth media. Similar observations were reflected for 5 min and 8 min exposure. Corresponding to *E. coli* enumeration, overlapped electropherograms are shown in Figure 4B. While the 0 min exposure sample showed a distinct peak, the 2 min exposure sample showed a slight shift in peak, which was further decreased in the 5 min exposure. In the 8 min samples, which showed no *E. coli* recovery, the electropherogram reflected an almost negligible peak (Figure 4B). 

### 2.3. Comparison between Automated Electrophoresis and Qubit Fluorometer Measurements

**Figure 5:**
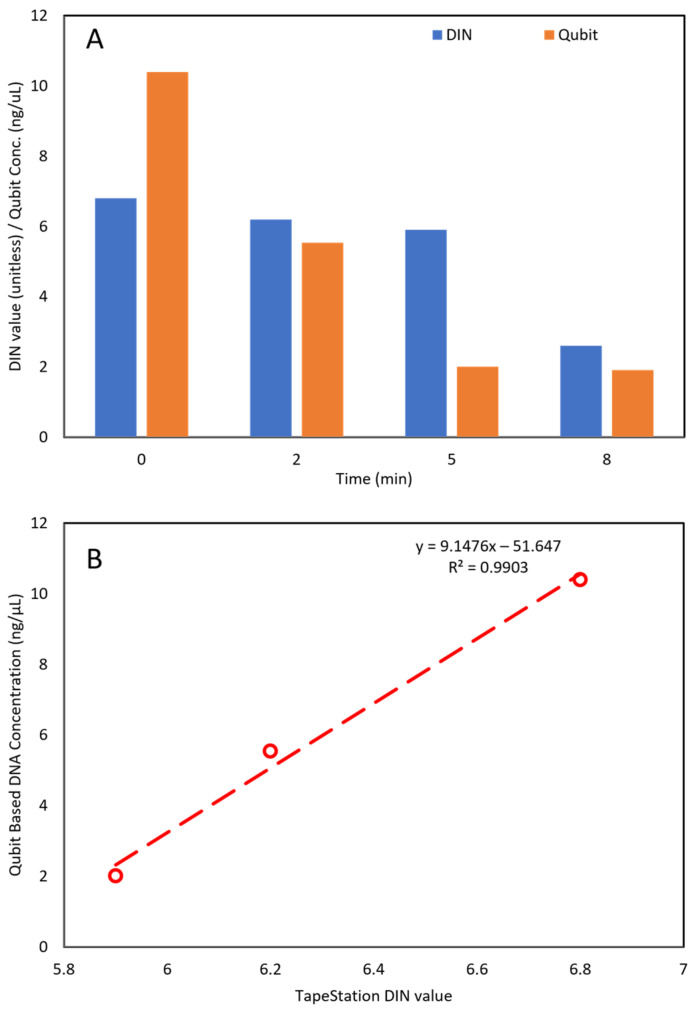
Results of the microwave exposure study using Qubit and TapeStation tests. Comparison of DNA degradation measurements between Qubit and TapeStation is shown: (**A**) shown DIN values were obtained from TapeStation; and DNA concentration in ng/µL was obtained from Qubit. (**B**) Shows linear relationships between DIN- and Qubit-based DNA concentrations.

A comparative analysis during the microwave exposure study was performed using two different methods: (1) automated electrophoresis; and (2) Qubit fluorometer measurements. Genomic DNA was quantified using both the 4200 TapeStation system and Qubit. The genomic DNA screen tape used in TapeStation uses fluorescent dye (specifically designed for double-stranded DNA). Comparative observations and results are shown in Figure 5. The Qubit method-based genomic DNA analysis of the control *E. coli* sample (without microwave exposure) resulted in a DNA concentration of 10.4 ng/µL. In this sample, the Qubit-based 10^00^ concentration was slightly higher than the TapeStation-based DIN. In the 2 min microwave exposure time, genomic DNA concentration analysis based on the Qubit method showed that the DNA concentration was reduced from 10.4 ng/µL to 5.54 ng/µL (about 46.7% reduction). In the 5 min exposure, the genomic DNA concentration (Qubit) was reduced by 80.6%. Further *E. coli* exposure to microwaves for 8 min resulted in genomic DNA degradation from 10.4 ng/µL to 1.91 ng/µL (81.6% reduction). The bar graph in Figure 5A shows the quantity of genomic DNA obtained from Qubit fluorometer and corresponding DIN values from TapeStation. The reduction in genomic DNA concentrations was proportional to the exposure time. At the end of 8 min of exposure time, genomic DNA was substantially low, and at this level of genomic DNA, there was no recovery of *E. coli* in modified mTEC agar media plates (Figure 4A). The relationship between genomic DNA integrity and genomic DNA concentrations was determined and results are shown in Figure 4B. The DINs of genomic DNA obtained from TapeStation 4200 for samples exposed to microwaves (0 min, 2 min, and 5 min) were plotted against genomic DNA concentrations obtained using the Qubit fluorometer. Results showed a strong correlation between DIN and DNA concentrations (*R*^2^ = 0.99). 

The findings of this study could improve our existing understanding of microwave radiation impacts on DNA integrity and the effects of microwave radiation on bacteria. The application of heat produced by microwave radiation is found to be useful in the removal of bacteria and moisture from food products. Previous studies have shown that microwave radiation is able to reduce airborne *E. coli* [23]. In terms of moisture removal, microwave-based heating accelerates drying and the moisture removal process. Conventional drying is relatively slower than microwave-based drying, and it has been tested in various food items including the removal of moisture from green peas [24]. The impacts of microwaves vary depending on the food items, and the drying of high-value products such as raisins by microwaves was relatively faster and efficient [25]. Results showed that microwave radiation facilitates unique heating characteristics, which creates better conditions for the removal of moisture [26]. In addition to drying, microwave-based heating produces food products which are pathogen-free, and the findings of this study substantiate previous results on the impacts of microwaves on bacteria removal. Further, previous experiments showed that aflatoxin B1 (carcinogens) could be reduced by microwave radiation [27]. In our study, we evaluated the role of microwave exposure on *E. coli* using a series of methods such as *E. coli* enumeration by a modified mTEC method and *E. coli* genomic DNA degradation measurement by automated gel electrophoresis and Qubit fluorometers, which is yet to be reported. 

While microwave radiation reduces the viability of bacteria such as *E. coli*, the impacts of microwaves on DNA are yet to be fully understood. Previous studies suggested that the resonant absorption of microwave radiation occurs in DNA, which may cause damage to DNA [28]. Microwave radiation is shown to inhibit protein, DNA, and RNA, and depending upon the field strength, frequencies, wave forms and exposure time, the impacts of microwaves on DNA change [29]. In a previous study [30], results showed that exposure to a low intensity of microwaves can induce oxidative stress and lead to DNA damage.

To evaluate DNA degradation, gel electrophoresis is used often, and previous studies have observed DNA degradation under various conditions such as temperature, time, and salt concentrations using gel electrophoresis [31,32]. At an elevated temperature, DNA degrades substantially [33,34]. In addition to a warmer temperature, alkaline conditions are known to accelerate environmental DNA degradation [35]. In ambient water bodies, water temperature is known to accelerate DNA degradation [36]. Environmental DNA degrades faster in ambient conditions and warmer conditions, and results also show that the DNA degradation is slower in salty water compared to purified water [36]. 

In this study, we applied automated gel electrophoresis to evaluate genomic DNA integrity in environmental samples after microwave exposure. Results are promising in terms of using microwaves for controlling bacterial pollution and degrading the DNA of pathogens and pathogenic bacteria. In microbiological studies, it is reported that microbial cell death is caused by both the heat produced by microwave radiation and the microwave electric field [29,37]. During microwave radiation, genomic DNA is fragmented, which degrades DNA. On the other hand, studies have shown that microwave-based fragmentation could be useful for next-generation sequencing (NGS) shotgun library preparation [38]. 

This study provides in-depth insight into the impacts of microwaves on bacteria and the genomic DNA of *E. coli*. Because of microwaves’ ability to kill bacteria, recently, microwave-based sterilization has been seen as promising due to speed and energy efficiency [39,40]. In a sterilization study, an aqueous solution of DNA was extracted from *E. coli*, the solution was exposed to microwave radiation (8–12 GHz), and results showed that *E. coli* DNA absorbs microwaves substantially [41]. While assessing reductions in *E. coli* colonies, previous studies have shown that approximately a 9 log reduction in *E. coli* can occur, when samples are exposed to microwave radiation [42]. Microwave sterilization can be integrated with steam-based sterilization and results showed that hybrid sterilization produces a 77% higher heat efficiency [40]. Microwave-assisted thermal sterilization is also used to produce ready-to-eat meals with extended shelf-life [43]. Microwave-assisted sterilization can increase nutrient retention and reduce the undesirable effects of heat during conventional heat-based sterilization [42,44]. This study particularly focused on environmental *E. coli* isolated from dairy manure for evaluating the impacts of microwaves on bacterial elimination. *E. coli* was responsible in multiple food-related outbreaks and many of these outbreaks were linked with livestock manure *E. coli* [45,46,47]. 

Microwave-assisted electromagnetic radiation provides numerous opportunities in the field of public health, sterilization, inactivation of spores in the food industry, wastewater disinfection, DNA extraction, and high-volume biological sample processing. Previous studies concluded that microwave radiation degrades DNA including human DNA [38,48]. By the use of an ultraviolet light source, a microwave discharge electrodeless lamp, the efficient disinfection of wastewater and inactivation of bacteria (Gram-positive B. subtilis and gram-negative *E. coli* inactivation) can be achieved, when wastewater is exposed to microwaves [49]. Research showed that microwave irradiation has a distinctive effect on fragmentation of DNA molecules, and electromagnetic energy from microwaves degrade DNA rapidly [38,50]. In a study, human genomic DNA (50 µg/mL) from a healthy blood sample was exposed to microwave radiation, and the results showed that the use of different microwave processing methods resulted in different patterns of fragment size distribution [38]. Previous studies showed that microwave exposure causes changes to DNA, and the extracted DNA of bacteria exposed to microwaves produces new bands on electrophoresis examination. A brighter and thicker DNA banding pattern was observed in DNA extracted from bacteria that was exposed to microwaves [51]. Recently, microwave-based technologies have been investigated for microbial load reduction because of the emphasis on microbial food safety, and research showed that microwave-based processes are economically feasible at an industrial scale [51,52]. To prevent the growth of malaria parasites, recently, microwave-based equipment has been developed to inactivate parasites and reduce the risks to public health [53,54]. In another study, a microwave-assisted cartridge was designed for the rapid diagnosis of M. tuberculosis (the causative agent of tuberculosis). The direct application of microwaves to a bacteria-containing sample resulted in the release of pathogen-specific DNA, which assisted in the diagnosis process [53].

We anticipate that the findings of this study will improve our existing understanding of microwave radiation on bacteria and genomic DNA and provide additional information to explore the application of microwave radiation in pathogenic bacteria control and pathogen DNA reductions. In terms of microwaves, there are numerous applications, for example, microwave radiation is useful in analytical and environmental chemistry, pretreatment, sample processing in research, food sterilization, drug discovery, large-scale drying, mineral processing, and chemical synthesis [55,56,57,58,59,60]. Recently, the application of microwave technology has been evaluated in manufacturing technology; road construction and de-icing; catalyst production; application as an absorbent in oil shale; solidification of copper; and polymer production [61,62,63,64,65,66]. The nature of this study, however, was limited to improving our existing understanding of microwave radiation’s impacts on environmental bacteria and evaluating the impacts of microwave levels on DNA integrity. High-throughput methods such as automated gel electrophoresis were used to determine the DNA integrity of *E. coli*, when samples were exposed to various levels of microwave radiation. The findings are significant when evaluating the scope of microwaves’ applications for ensuring the microbial safety of food products and developing the protocols for testing the quality of DNA in post-treated samples. 

## 3. Conclusions

In this study, automated gel electrophoresis was used to evaluate the impacts of microwave radiation on genomic DNA integrity and bacterial cells. Automated gel electrophoresis, TapeStation 4200, was used for rapid DNA quality tests. Further, genomic DNA concentrations were monitored using a Qubit fluorometer. Results showed no recovery of *E. coli* in modified agar growth media, when DNA integrity (i.e., DIN) was reduced by 8.8%. The reduction in DIN by 8.8% resulted in 46% genomic DNA losses monitored by the Qubit fluorometer. In 5 min of microwave exposure, DNA integrity was reduced by 13.2%; however, this level of microwave exposure resulted in 80% losses of genomic DNA concentrations. Beyond 2 min of microwave exposure, *E. coli* was not detectable/recoverable in agar plates. Results showed that the DIN value was highly correlated with the Qubit-based genomic DNA concentrations, and DIN can be used to determine the genomic DNA integrity and viability of *E. coli* in environmental and food-related samples. 

## 4. Material and Methods

### 4.1. E. coli Isolation and Membrane Filtration Method

**Figure 6:**
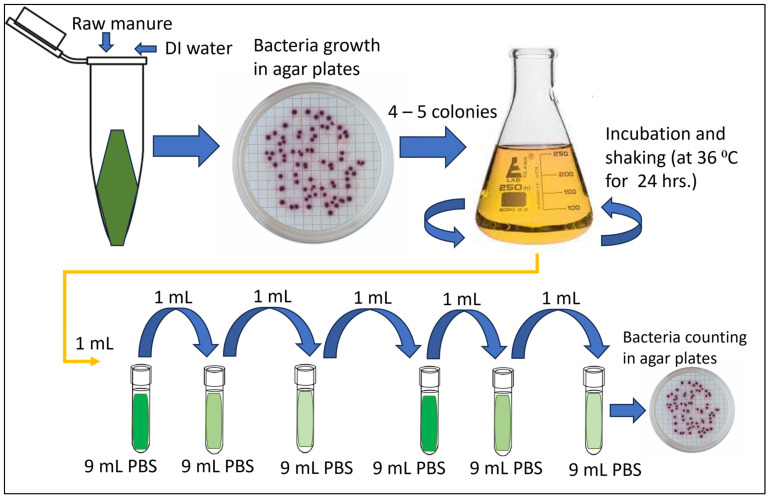
The isolation of *E. coli* from fecal samples and subsequent detection of *E. coli* levels using modified mTEC agar.

**Figure 7:**
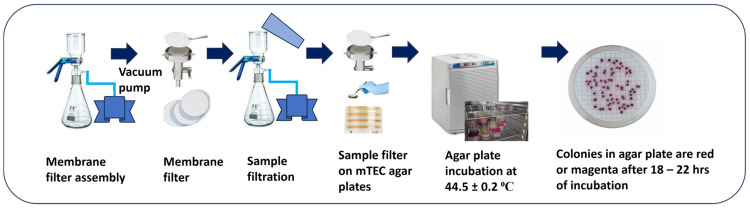
The enumeration of *E. coli* using the membrane filtration method and incubation–based viable cell detection in modified mTEC agar.

The membrane filtration method is a procedure for enumerating *E. coli* from fluid samples. It quantifies *E. coli* in fluid samples within 24 h. To isolate *E. coli*, we diluted 1 g of dairy manure samples with 9 mL of phosphate buffer saline (PBS) (Figure 6), and this solution was further serially diluted to 10^−3^ levels. The diluted sample (100 µL) was used for detecting *E. coli* using the membrane filtration method (Figure 7). The diluted sample was filtered through a membrane filter (0.45 µm), and the filter was placed in a modified mTEC agar plate. Modified mTEC agar is used for enumerating and isolating *E. coli* in liquid, and it is a selective culture medium for chromogenic enumeration of thermotolerant *E. coli*. The agar plate with the filter was placed in an incubator (44.5 °C) for 18–22 h, which allows for the recovery/growth of *E. coli* in agar plates. The magenta colonies in the agar plate (Figure 6) were enumerated as *E. coli* colonies using a handheld colony counter with a pen. To count colonies in agar plates, we procured an eCount colony counter from Sigma-Aldrich (St. Louis, MO, USA). To culture bacteria, BD Difco^TM^ (1 Becton Drive Franklin Lakes, NJ, USA) Modified mTEC Agar was obtained from Fisher Scientific (Waltham, MA, USA). Gridded membrane filters, with a 0.45 µm pore size (S-pak), were obtained from Millipore. This membrane filter is designed to give complete retention and enhanced recovery of fecal coliform bacteria in liquid samples such as water. The procedure is well accepted by U.S. EPA Standard methods [67] and meets the ASTM specification for membrane filtration in drinking water analysis. 

### 4.2. Genomic DNA Extraction from Fecal Thermotolerant E. coli

**Figure 8:**
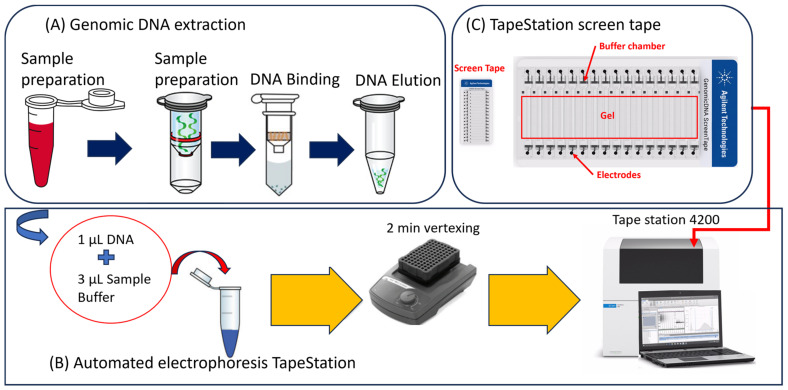
The extraction of genomic DNA of *E. coli* (**A**) and the application of automated gel electrophoresis for evaluating the integrity of DNA of *E. coli* (**B**,**C**).

To extract the genomic DNA, we used the Quick-DNA^TM^ fecal/soil microbe microprep kit, which was purchased from Zymo Research. This rapid kit allows for the isolation of inhibitor-free, PCR-quality DNA from Gram-positive and -negative organisms including bacteria, fungi, algae, and protozoa in soil and fecal samples. This method involves bead beating and a spin column, and the kit contains BashingBead tubes, buffer, genomic lysis buffer, genomic DNA wash buffer, DNA elution buffer, prep solution, spin filters, spin columns, and collection tubes. The DNA extraction workflow is shown in Figure 8A. This method allows for the extraction of high-quality DNA, it recovers genomic DNA up to and above 40 kb, and it recovers up to 5 µg total DNA, which can be eluted through 20 µL DNA elution buffer per sample. The extraction procedure requires a microcentrifuge, vortex, and cell disrupter. In order to extract DNA, 250 µL of sample was added to the BashingBead Lysis Tube, and it was mixed with 750 µL BashingBead Buffer. The BashingBead Lysis tube was centrifuged at ≈10,000× *g* for 1 min. The supernatant (400 µL) was transferred to a Zymo-Spin filter in a collection tube and centrifuged at 8000× *g* for 1 min. Subsequently, the Zymo-Spin filter was discarded and 1200 µL genomic lysis buffer was added to the filtrate in the collection tube. After mixing, 800 µL of the mixture was transferred to a Zymo-Spin IC column in a collection tube and centrifuged at 10,000× *g* for 1 min. After discarding the flow through from collection tube, 200 µL DNA pre-wash buffer was added to the Zymo-Spin IC column and centrifuged at 10,000× *g* for 1 min. DNA wash buffer was added to the Zymo-Spin IC column and centrifuged at 10,000× *g* for 1 min. The Zymo-Spin IC column was transferred to a clean 1.5 mL microcentrifuge tube and 20 µL DNA elution buffer was added directly to the column matrix. After centrifuging at 10,000× *g* for 30 s, the Zymo-Spin µHRN filter was placed in a clean collection tube, and 600 µL prep solution was added and centrifuged at 8000× *g* for 3 min. Eluted DNA was transferred to a Zymo-Spin µHRC filter in a clean 1.5 mL microcentrifuge tube and the tube was centrifuged at exactly 16,000× *g* for 3 min. Subsequently, the filtered DNA was used for downstream applications. 

### 4.3. Automated Genomic DNA Integrity Evaluation of E. coli

Automated genomic DNA extraction was performed using the Agilent 4200 TapeStation system (Figure 8B), which provides DNA quantification, qPCR work flow, and quality control for next-generation sequencing. The system separates nucleic acids by means of electrophoresis and provides a fully automated analysis of DNA quality, gel images, and electropherogram. The system can detect fluorescently stained double-stranded DNA and cell-free DNA. Based on the analysis, the system provides the DNA integrity number (DIN), as a measure of genomic DNA integrity, and DNA base pair (bp) size and distribution. The method requires 1–2 µL of DNA samples for high-sensitivity analysis. TapeStation-based analysis requires screen tape (Figure 8C), 2 µL DNA sample, 2 µL DNA sample buffer, the ladder, and vortexer. To prepare the sample for the analysis, we used 2 µL of a genomic DNA sample, which was mixed with 1 µL of sample buffer in a well plate. Similarly, the ladder well was prepared by mixing 2 µL of ladder and 1 µL of sample buffer. After preparing well tube for the ladder and samples, the well tube strip cap was closed to seal the wells, the well strip was spun at 2000 rpm for 2 min, and subsequently the well tube/plate was placed in the TapeStation. Caps of the tube strip were removed before loading the well strip in the machine. We used genomic DNA ScreenTape (Figure 8C) for analyzing genomic DNA from 200 to >60,000 bp. Data analysis was conducted using TapeStation 4200 Analysis Software, which provided gel images and electropherograms. 

### 4.4. Microwave Experiment for E. coli DNA Degradation and Genomic DNA Monitoring

**Figure 9:**
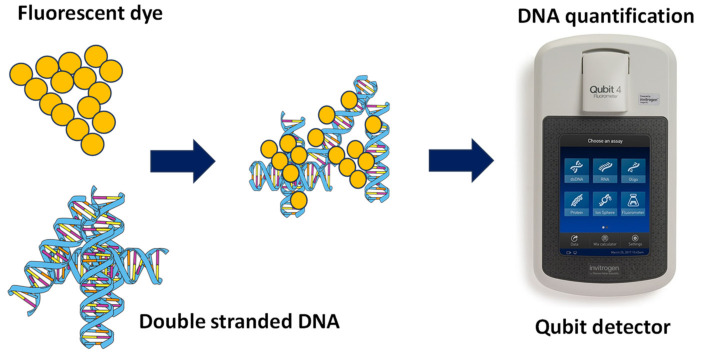
Mixing of fluorescent dye and genomic DNA and the application of the Qubit fluorometer for the quantitative analysis of the genomic DNA of *E. coli*.

To conduct microwave-based degradation experiment, 10 mL of *E. coli* culture was exposed to microwaves. This culture was grown overnight at 36 °C (Figure 1), and *E. coli* was extracted from dairy manure collected from three dairy farms located in California. The experiment was conducted in triplicate (3 beakers). All three beakers were kept in the microwave cavity, which receives microwaves generated by a magnetron (4.5 kW). The magnetron generates microwaves using the interaction of the electron stream with the magnetic field. These microwaves produced by the magnetron heat the liquid. The first sample was collected prior to microwave radiation exposure. The second sample was collected after 2 min of microwave exposure. The third sample was collected after 5 min of microwave exposure, and the fourth sample was collected after 8 min of microwave exposure. After exposing *E. coli* culture samples to microwaves, DNA was extracted from the sample and DNA testing was conducted using an automated TapeStation 4200 (Figure 8) and Qubit 4 fluorometer (Figure 9). 

## Data Availability

Data are contained within the article.
